# Femtosecond Laser Fabrication of Stable Hydrophilic and Anti-Corrosive Steel Surfaces

**DOI:** 10.3390/ma12203428

**Published:** 2019-10-20

**Authors:** Christina Lanara, Alexandros Mimidis, Emmanuel Stratakis

**Affiliations:** 1 Foundation for Research and Technology-Hellas (F.O.R.T.H.), Institute of Electronic Structure and Laser (I.E.S.L.), 70013 Heraklion, Greece; lanara@iesl.forth.gr (C.L.); amimidis@iesl.forth.gr (A.M.); 2 Department of Materials Science and Technology, University of Crete, 70013 Heraklion, Greece

**Keywords:** femtosecond laser processing, steel, wettability, anticorrosion

## Abstract

We report on a novel single-step method to develop steel surfaces with permanent highly hydrophilic and anti-corrosive properties, without employing any chemical coating. It is based on the femtosecond (fs) laser processing in a saturated background gas atmosphere. It is particularly shown that the fs laser microstructuring of steel in the presence of ammonia gas gives rise to pseudoperiodic arrays of microcones exhibiting highly hydrophilic properties, which are stable over time. This is in contrast to the conventional fs laser processing of steel in air, which always provides surfaces with progressively increasing hydrophobicity following irradiation. More importantly, the surfaces subjected to fs laser treatment in ammonia exhibit remarkable anti-corrosion properties, contrary to those processed in air, as well as untreated ones. The combination of two functionalities, namely hydrophilicity and corrosion resistance, together with the facile processing performed directly onto the steel surface, without the need to deposit any coating, opens the way for the laser-based production of high-performance steel components for a variety of applications, including mechanical parts, fluidic components and consumer products.

## 1. Introduction

The ultrafast laser processing of solid surfaces has been established as a reliable method to modify a material to selectively control the wetting [1,2,3,4], optical [5,6] and tribological properties [7]. In the special case of wetting properties, inspiration has often been drawn from surface patterns found in natural archetypes, which are mimicked by ultrashort pulsed laser structuring [1,8]. Among the targeted extreme wetting properties, superhydrophilicity has received significant attention due to its numerous potential applications [9] in self-cleaning [10], drag reduction [11], heat management [12], fluid transport [13] and biological cell adhesion [14].

Due to its significant relevance in a plethora of industrial applications, steel has often been the focus of research concerning materials’ surface functionalization. Although many works have been reported on the modification of steel wettability via conventional laser texturing, the wetting properties attained are unstable and always switch from a superhydrophilic to a highly hydrophobic state within a few days after irradiation [15,16,17]. This is attributed either to carbon contamination of the laser-treated surfaces [18] or to chemical composition instability [19]. It is evident that such evolution of the surface chemistry, energy and thus wettability substantially limits the potential applications of laser-processed steel [1,8]. In this context, it is a challenge to maintain stable hydrophilic characteristics, in the long term, on a metallic surface. Recently, Rajab et al. showed that the surface chemistry of laser-textured steel can be rendered superhydrophilic for a long time upon the post-processing deposition of a hydrophilic coating. This approach, however, requires an additional surface coating step to sustain surface hydrophilicity [20]. Therefore, to date, there is no report on the realisation of a stable hydrophilic metallic surface, without using any chemical coating, via a simple, one-step texturing process. 

In this work we first demonstrate a facile, single-step method to fabricate stable hydrophilic steel surfaces based on femtosecond (fs) laser processing in the presence of a saturated background gas atmosphere [21]. Although laser texturing is conventionally performed in ambient air, the presence of a surrounding medium, i.e., a background gas or liquid, affects both the attained surface features and chemistry [22,23,24]. In particular, it is shown that the fs laser microstructuring of steel in the presence of ammonia gas gives rise to pseudoperiodic arrays of microcones (MCs) exhibiting highly hydrophilic properties, which are stable over time, at least for a five-month period. Energy dispersive X-ray spectroscopy (EDS) analysis reveals that the observed hydrophilicity is attributed to the termination of the laser-treated surface with nitrogen groups. The findings are compared to the conventional laser texturing of steel in ambient air using the same laser beam parameters. It is concluded that the interplay between increased surface roughness and polar surface chemistry enhances the hydrophilicity of the attained surfaces. At the same time, it is found that the laser-textured surfaces in ammonia exhibit remarkable corrosion resistance. Our method not only provides a dual functionality in laser-textured steel surfaces, but it is also simple, without the need of any additional surface coating. As a consequence, it could find a broad range of practical applications in the steel industry. 

## 2. Materials & Methods

### 2.1. Material

Commercially polished steel alloy samples 40CrMnMoS8-6 (1.2312), supplied by ML Engraving (Onore, Italy), were used in this study. In particular, the chemical composition of the material consisted of (0.35–0.48) wt % C, (0.30–0.50) wt % Si, (1.40–1.60) wt % Mn, (0.03) wt % P, (0.05–0.10) wt % S, (1.80–2.00) wt % Cr and (0.15–0.25) wt % Mo. Prior to laser processing, the samples were cleaned in an ultrasonic ethanol (Elma Schmidbauer GmbH, Singen, Germany) bath for 10 min. 

### 2.2. fs Laser Surface Structuring

An Yb:KGW (ytterbium-doped potassium gadolinium tungstate) (Light Conversion, Vilnius, Lithuania) laser source was used to produce linearly polarized pulses of 170 fs duration, 1 KHz repetition rate and 1026 nm central wavelength (Supplementary Materials, Table S1). The Ti: Sapphire laser system is a high repetition rate femtosecond laser system based on the chirped pulse amplification (CPA) technique, which uses directly diode-pumped Yb:KGW crystals as the active medium. The automated second/third harmonic module for the Ti: Sapphire laser system offers the possibility to choose between three laser wavelengths (1030 nm, 515 nm and 343 nm) by sending a command via computer (or Remote Control Module (RCM). The switching between wavelengths in the harmonic module is implemented by means of switchable mirrors, which are controlled by two servo motors. The fundamental emission from the laser is directed through a periscope of two mirrors to the output of the harmonics module. The polarization of the output at 1030 nm is horizontal.

**Figure 1:**
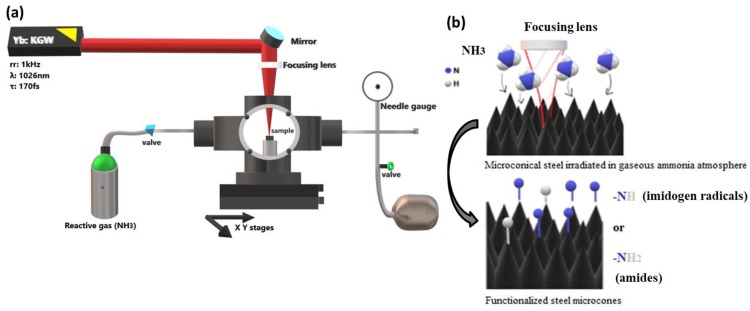
(**a**) Schematic of the experimental ultrashort laser setup used in this study. Experimental procedure of the fabrication of the functionalized micro/nanopatterned steel substrates using ammonia gaseous atmosphere during the irradiation procedure; (**b**) Schematic of micropatterned substrates during and after ultrashort pulsed laser processing under gaseous ammonia atmosphere.

Figure 1a presents the experimental setup used for the laser processing. The whole process took place in a vacuum chamber evacuated down to a residual pressure of approximately 10^−2^ mbar by means of a mechanical oil pump. The attached micro valve system, coupled with a needle gauge, enabled the precise backfilling of the background NH_3_ gas. In our case, the NH_3_ pressure used was equal to 800 mbar. The sample was placed inside the vacuum chamber on a metallic base. The laser beam, entered into the processing chamber through a quartz entrance window, was focused by a 15 cm focusing plano convex lens onto the sample surface. For areal scanning, the chamber was placed on a computer-driven, high-precision, X-Y translation stage, enabling the sample displacement with respect to the laser beam with a scan velocity of 500 μm/s and a line scan separation of 20 μm. For this study, areas of 5 mm × 5 mm were fabricated. The irradiation process was visualized through a Plexiglas window, which was laterally mounted on the vacuum chamber. Following laser treatment, the samples were cleaned in an ethanol ultrasonic bath for 15 min, dried by blowing nitrogen gas and stored in ambient air.

### 2.3. Corrosion Test 

The evaluation of the corrosion of the untreated and laser-processed steel surfaces comprised the immersion of the samples into an aqueous salt dilution (NaCl/H_2_O:50 gr/L) at 35 °C for two hours. For the aqueous salt dilution synthesis, solid salt (NaCl) (double-distilled water and a glass beaker were used. The heating procedure of the dilution was carried out via a hot plate equipped with an electronic thermometer. Following the process, the samples were dried by blowing nitrogen gas and were subsequently imaged using an optical microscope (Leica DMR) (Leica microsystems, Wetzlar, Germany.

### 2.4. Characterization of Micropatterned Steel Substrates

The laser-irradiated samples were morphologically characterized by field emission scanning electron microscopy (SEM; JEOL 7000) (JEOL (Europe) BV, Zaventem, Belgium). An image-processing algorithm (ImageJ, National Institutes of Health, Bethesda, MD, USA) was implemented in order to determine the topological characteristics of both air- and ammonia-structured MCs, including the roughness ratio, height and tip radiuses, using side- and top-view SEM images. More specifically, both air- and ammonia-structured surfaces were considered as conical frustum. The corresponding roughness ratios were calculated by dividing the actual, unfolded surface area of spikes with the total irradiated area. The mean value of the roughness ratio was calculated from statistics performed at 10 individual substrates. Besides this, energy-dispersive X-ray spectroscopy (EDS) (JEOL (Europe) BV, Zaventem, Belgium) was used to characterize the chemical composition attained. The surface wettability was investigated via static contact angle (CA) measurements, performed in an automated tensiometer. Using a microsyringe (Brand, Wertheim, Germany), a 3 μL distilled, deionized Millipore water droplet was gently positioned on each surface tested and images of the droplet profile were captured to measure the angle formed at the liquid–solid interface. The mean CA value was then calculated from three individual measurements. 

## 3. Results 

### 3.1. Surface Wettability

The micropatterned steel surfaces were produced upon the fs laser structuring of polished steel wafers in both air and NH_3_ atmosphere.

Depending on the laser fluence used, this technique offers the advantage of the patterning of steel with periodic arrays of structures ranging from submicron to micron sized. The reason for the selection of the fluence value of 0.73 J/cm^2^ was the requirement of high-roughness surfaces. More specifically, we have previously shown that for laser fluences larger than 0.5 J/cm^2^ and lower than 1 J/cm^2^, the formation of MCs takes place on steel alloy samples [1]. Regardless of the irradiation environment, the processed surfaces comprised submicron-sized ripples at low laser fluences, while, at the higher ones used, quasi-periodical arrays of MCs were formed.

**Figure 2:**
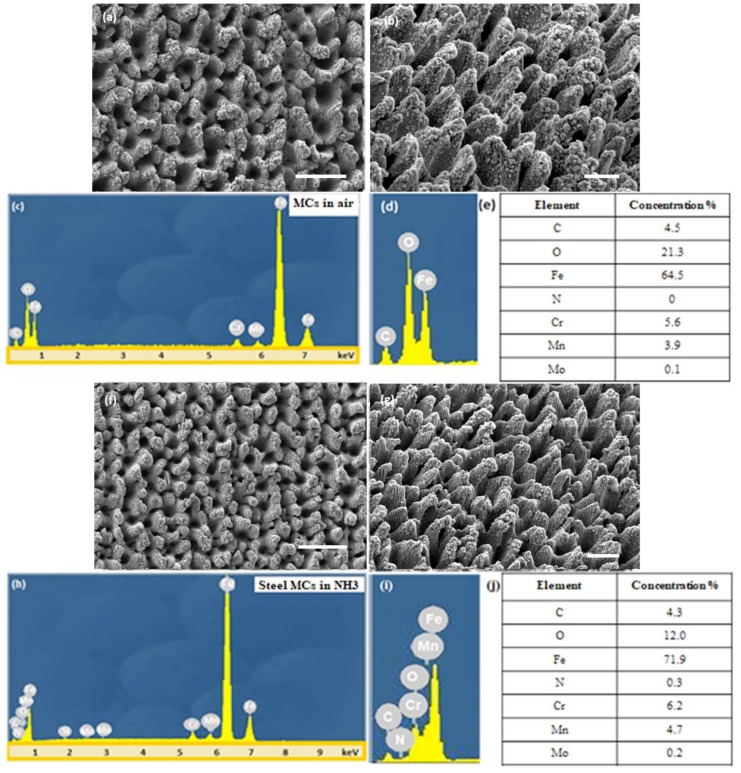
Top-view (**a**) and 45°-tilted (**b**) SEM images of steel surfaces fabricated in air at a fluence of 0.73 J/cm^2^ (scale bars: 50 μm and 20 μm, respectively); (**c**, **d**) EDS spectra from the area shown in (a). The corresponding elemental analysis is shown in (**e**); Top-view (**f**) and 45°-tilted (**g**) SEM images of steel surfaces fabricated in NH_3_ atmosphere at a fluence of 0.73 J/cm^2^ (scale bars: 50 μm and 20 μm, respectively); (**h**, **i**) EDS spectra from the areas shown in (**f**). The corresponding elemental analysis is shown in (**j**).

Figure 2 presents typical SEM images of the respective surface morphologies attained at the highest laser fluence used of 0.73 J/cm^2^. It can be observed that in all cases the MCs formed exhibited a hierarchical morphology, in the sense that the primary micro-sized spike structures produced exhibited a secondary nano-sized roughness. 

The flat polished steel surface appeared at a mean roughness of a few tens of nanometers. Laser structuring in the presence of NH_3_ gas atmosphere was shown to reduce the overall surface roughness, compared to laser treatment in air, upon using identical laser beam characteristics. As a result, the MCs fabricated in gaseous NH_3_ were shorter and smaller than those fabricated in air (Figure 2a,b,f,g). The respective heights were (14.2 ± 5.7) μm and (11.6 ± 4.4) μm, while the tip radii were (7.1 ± 2.8) μm and (6.5 ± 2.2) μm for air and NH_3_ ambients, respectively. The corresponding roughness ratios calculated were equal to 2.1 for the ammonia-treated and 2.3 for the air-treated samples. This size difference can be explained on the basis of the confinement and shielding effect of plasma under different environmental conditions [25]. The NH_3_ gas was found to play an important role in the MCs’ fabrication process, as it not only affects the size, but also the chemistry of the structures attained. This was indicated by the elemental analysis performed via EDS, showing an emergence of a characteristic nitrogen peak in the samples irradiated in NH_3_ atmosphere (Figure 2h). At the same time, the oxygen content became lower in the NH_3_-processed samples, compared to the air-processed ones (Figure 2d,i). Further analysis via X-ray Photoelectron Spectroscopy is in progress to elucidate the exact nitrogen groups terminating the surface, for example amides (-NH) of imidogen (-NH_2_) groups.

**Figure 3:**
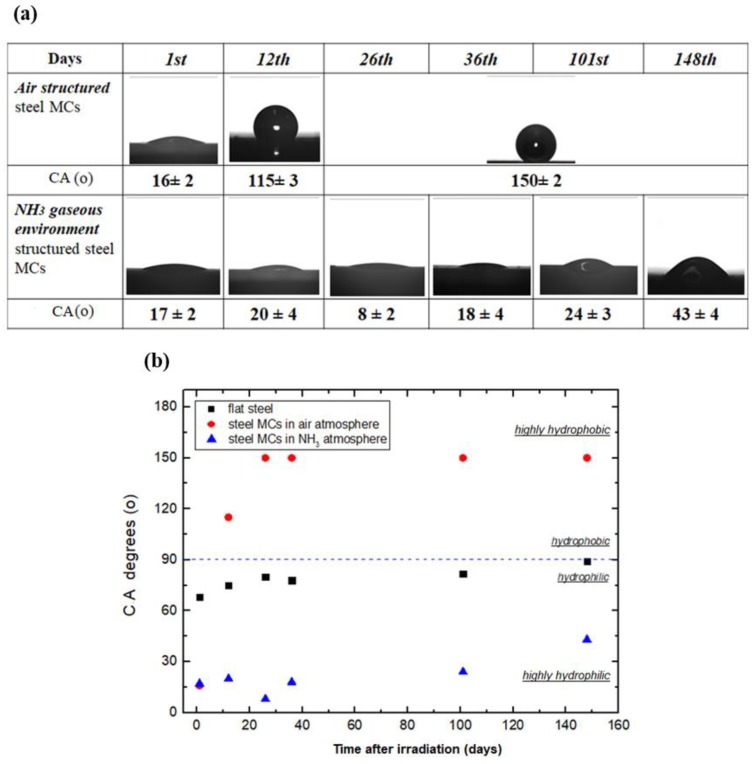
(**a**) Evolution of the profile of a water droplet placed onto laser-fabricated steel surfaces in air (top) and NH_3_ (bottom) atmosphere, respectively, as a function of the time after irradiation; (**b**) Contact Angle (CA) evolution for 148 measurement days of flat (black spots), air-structured (red spots) and ammonia-structured (blue spots) steel surfaces.

Figure 3a depicts the droplet profiles and the corresponding CA values measured on the laser-functionalized steel surfaces as a function of time after irradiation. Contrary to the samples processed in air, which became highly hydrophobic within a few days of irradiation [2,18], the surfaces structured in the presence of NH_3_ gas maintained their high hydrophilicity for at least five months after the laser treatment. Figure 3b compares the CA evolution over time for the flat, air-processed and NH_3_-processed steel samples, respectively. It was noticeable that laser processing in NH_3_ gave rise to permanent surface hydrophilicity, which was even higher than the untreated steel surfaces. What was also observed was that following 100 days from laser treatment, there was a slight decrease of hydrophilicity in both flat and NH_3_-treated samples, possibly attributed to surface contamination or to absorbed airborne hydrocarbon molecules [18].

Nitridation of metals or their derivatives by using N_2_ or NH_3_ is a classical method of preparing nitrides [26]. For example, aluminium nitride can be obtained by heating the metal with NH_3_ or N_2_ at a high temperature. The microstructuring of steel by fs lasers under NH_3_ atmosphere was chosen as a method of surface nitridation because ammonia plasmas produce large quantities of atomic nitrogen (N) and hydrogen (H), as well as NH_2_ groups that readily interact with steel. In particular, reactions such as photo-induced dissociation or plasma-induced dissociation of ammonia, e.g., NH_3_~NH + H_2_, were observed by optical spectroscopy, and thus the reaction Steel + NH~SteelN + H, either in the gas phase or on the surface, is feasible [26]. Besides this, NH_3_ plasma is used to etch and chemisorb on steel [27,28]. However, heating at approximately 1000 °C may also initiate a thermal reaction. This suggests that laser heating could cause a reaction, giving rise to a strong and stable covalent bonding of nitrogen on steel surfaces [29]. At the same time, ammonium dilutions tend to etch metal surfaces [27], leading to a strong and stable covalent bond of nitrogen on them [29].

### 3.2. Surface Corrosion Properties

During corrosion, metals react with the nonmetallic elements of their environment and produce chemical compounds [30]. In our case, air-processed and NH_3_-processed steel samples were tested for their anti-corrosion behavior upon their reaction with an aqueous salt dilution (NaCl/H_2_O:50 gr/L) at 35 °C for two hours, which is a very reliable protocol [31].

**Figure 4:**
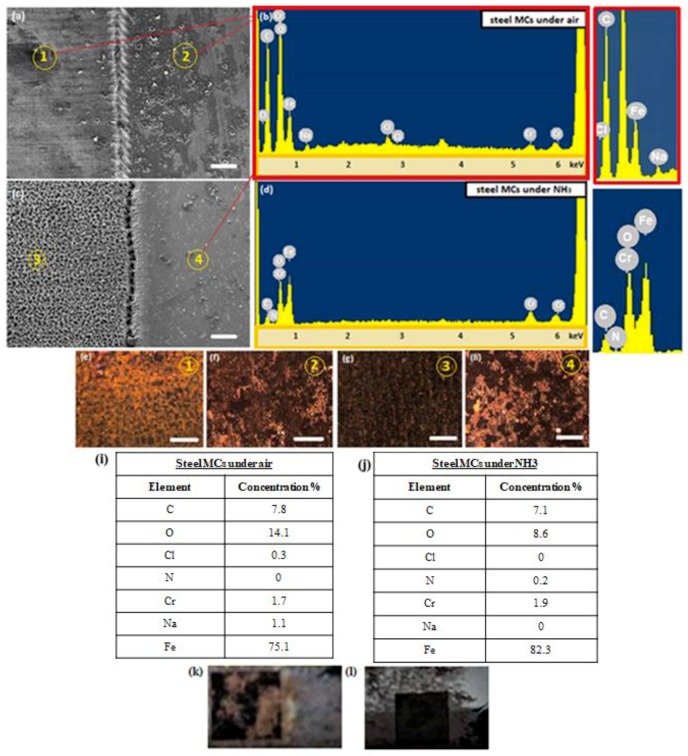
(**a**) Top-view SEM image of steel micro-cones (MCs) fabricated in air following the corrosion test (scale bar: 100 μm); (**b**) EDS spectra from regions 1 and 2 shown in (a). The corresponding elemental analysis is shown in (**i**); (**c**) Top-view SEM image of steel MCs irradiated in NH_3_ (scale bar: 100 μm); (**d**) EDS spectra from region 3 shown in (c); The corresponding elemental analysis is shown in (**j**); (**e**–**h**) Microscope images of regions 1–4 shown in the SEM images (a,c) (scale bar: 100 μm); Photograph of laser-treated steel sample in air (**k**) and ammonia (**l**); The black square corresponds to the structured area.

Figure 4 presents the characteristic surface morphologies obtained after the corrosion test, characterised by SEM (a,c), confocal (e–h) and optical (k,l) microscopy. It can be observed that for the air structured steel surfaces, a large number of salty patterns were formed (Figure 4a,e,f), on both micro-structured (region 1) and untreated (region 2) areas. On the contrary, the NH_3_-structured steel surfaces did not show any salty patterns on the micro-structured area (region 3 of Figure 4c,g), although many of such patterns appeared on the untreated area of these samples (Figure 4h corresponding to region 4). The effect of corrosion was also examined by EDS elemental analysis, which confirmed the presence of sodium (Na) and chlorine (Cl) elements on the air-treated and untreated areas (Figure 4b,i). On the contrary, the steel substrates irradiated under NH_3_ gas did not present such elements in their respective EDS spectra (Figure 4d,j). These results were confirmed by the optical imaging of the samples, showing the absence of any oxidation layer in NH_3_-treated steel samples, contrary to the air-treated ones. 

The role of nitrogen in the mechanisms of local corrosion resistance and re-passivation was electrochemically investigated in austenitic stainless steels [32]. It was shown that nitrogen atoms, present at the boundary between the metal and the oxidation layer, effectively induced the re-appearance of passivation layer and, in this way, increased the localized corrosion resistance of this film. Further investigations, however, are required to figure out the exact mechanism of the anticorrosion behavior of laser-processed steel in a reactive ammonia atmosphere.

## 4. Conclusions

As it is shown, the fs laser microstructuring of steel in the presence of ammonia gas provides a dual functionality on steel, namely hydrophilicity and corrosion resistance. In particular, it is shown that the laser-treated steel surfaces exhibit highly hydrophilic properties, which are stable over time. At the same time, it is demonstrated that the surfaces subjected to fs laser treatment in ammonia exhibit remarkable anti-corrosion properties, contrary to those processed in air, as well as untreated ones. Our findings demonstrate the possibility of improving steel surface functionalities upon fs laser processing in the presence of a reactive gas and open the way for the production of high-performance steel components for various potential applications.

## Supplementary Materials

The following are available online at https://www.mdpi.com/1996-1944/12/20/3428/s1, Table S1: Parameters used for laser processing.
