# Supplementary material for: Femtosecond Laser Fabrication of Stable Hydrophilic and Anti-Corrosive Steel Surfaces
Source: Materials (Basel). 2019 Oct 20;12(20):3428. doi: 10.3390/ma12203428 (PMC6829529; doi:10.3390/ma12203428)

Electronic Supplementary Material (ESI) for Optical Materials Express.

Electronic Supplementary Information (ESI)

Table S1. Parameters used for laser processing

Laser processing parameters	
Wavelength λ (nm)	1026
Pulse duration τ (fs)	170
Repetition rate f (kHz)	1
Focus diameter $2w_o$ (µm)	100-110
Line separation Δy (µm)	20
Scan velocity v_x (mm/s)	0.5
Laser fluence (J/cm^2)	0.73
Laser power (mW)	170

ESI Figure 2. EDS spectra of the clean untreated steel surface (flat area)

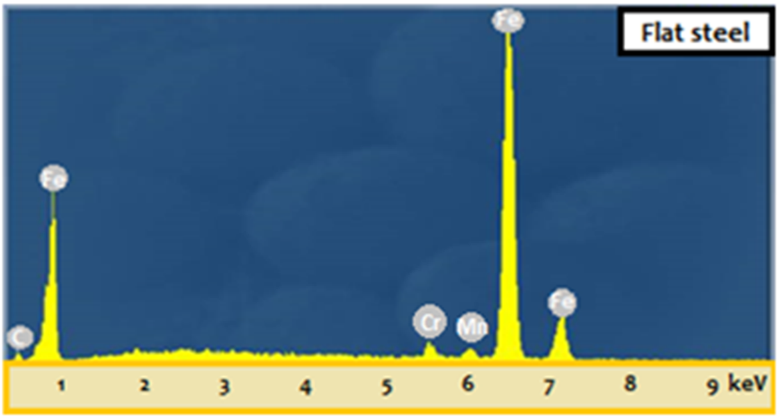

Supplement: Supplementary file 1 [file materials-12-03428-s001.pdf]
